# Identification of Multiple Cryptococcal Fungicidal Drug Targets by Combined Gene Dosing and Drug Affinity Responsive Target Stability Screening

**DOI:** 10.1128/mBio.01073-16

**Published:** 2016-08-02

**Authors:** Yoon-Dong Park, Wei Sun, Antonio Salas, Avan Antia, Cindy Carvajal, Amy Wang, Xin Xu, Zhaojin Meng, Ming Zhou, Gregory J. Tawa, Jean Dehdashti, Wei Zheng, Christina M. Henderson, Adrian M. Zelazny, Peter R. Williamson

**Affiliations:** a Laboratory of Clinical Infectious Diseases, National Institute of Allergy and Infectious Diseases, National Institutes of Health, Bethesda, Maryland, USA; b National Center for Advancing Translational Sciences, National Institutes of Health, Bethesda, Maryland, USA; c Protein Characterization Laboratory, Cancer Research Technology Program, Frederick National Laboratory for Cancer Research, Frederick, Maryland, USA; d Microbiology Service, Department of Laboratory Medicine, Clinical Center, National Institutes of Health, Bethesda, Maryland, USA; e Section of Infectious Diseases, Department of Medicine, University of Illinois at Chicago College of Medicine, Chicago, Illinois, USA

## Abstract

*Cryptococcus neoformans* is a pathogenic fungus that is responsible for up to half a million cases of meningitis globally, especially in immunocompromised individuals. Common fungistatic drugs, such as fluconazole, are less toxic for patients but have low efficacy for initial therapy of the disease. Effective therapy against the disease is provided by the fungicidal drug amphotericin B; however, due to its high toxicity and the difficulty in administering its intravenous formulation, it is imperative to find new therapies targeting the fungus. The antiparasitic drug bithionol has been recently identified as having potent fungicidal activity. In this study, we used a combined gene dosing and drug affinity responsive target stability (GD-DARTS) screen as well as protein modeling to identify a common drug binding site of bithionol within multiple NAD-dependent dehydrogenase drug targets. This combination genetic and proteomic method thus provides a powerful method for identifying novel fungicidal drug targets for further development.

## IMPORTANCE

Cryptococcosis is a neglected fungal meningitis that causes approximately half a million deaths annually. The most effective antifungal agent, amphotericin B, was developed in the 1950s, and no effective medicine has been developed for this disease since that time. A key aspect of amphotericin B’s effectiveness is thought to be because of its ability to kill the fungus (fungicidal activity), rather than just stop or slow its growth. The present study utilized a recently identified fungicidal agent, bithionol, to identify potential fungicidal drug targets that can be used in developing modern fungicidal agents. A combined protein and genetic analysis approach was used to identify a class of enzymes, dehydrogenases, that the fungus uses to maintain homeostasis with regard to sugar nutrients. Similarities in the drug target site were found that resulted in simultaneous inhibition and killing of the fungus by bithionol. These studies thus identify a common, multitarget site for antifungal development.

## INTRODUCTION

Cryptococcal meningitis, caused by the fungus *Cryptococcus neoformans*, results in approximately 600,000 deaths from 1,000,000 infections annually (1, 2). The disease is associated predominately with immunosuppressed individuals, such as those infected with HIV or immunosuppressed with transplant conditioning or cancer chemotherapy, but it can also occur in previously healthy individuals as well (3, 4). In countries with a high prevalence of HIV/AIDS, such as those in sub-Saharan Africa, *Cryptococcus neoformans* is one of the most common causes of meningitis (1). Administration of intravenous amphotericin B and flucytosine is the standard therapy regimen for cryptococcal meningitis patients (5); however, due to the renal toxicity and lack of oral formulations of amphotericin B and hematological toxicity of flucytosine, novel anticryptococcal drugs are sorely needed. Fluconazole is an important orally absorbed, nontoxic drug useful for prophylaxis and follow-up treatment after amphotericin B induction, but administration in the acute setting is associated with poor efficacy and a 90% mortality rate (6). The fungicidal activity of amphotericin B is thought to be critical for its efficacy in the acute setting, with the rate of fungal clearance in the cerebrospinal fluid (early fungicidal activity [EFA]) being an important discriminator between ineffective fungistatic therapies such as fluconazole and more effective fungicidal therapies such as amphotericin B (6). Thus, identifying new drug targets, especially those associated with fungicidal activity, has become a priority (7).

Previously, the parasitic drug bithionol was demonstrated to have fungicidal properties based on a high-throughput drug-repurposing screen of 1,280 pharmacologically active compounds against *Cryptococcus neoformans* (8). Bithionol is a diphenolic compound that, prior to the advent of praziquantel, was used extensively as an anthelmintic agent against pulmonary paragonimiasis for both individual and mass treatment in areas where paragonimiasis is endemic (9). The drug is well tolerated in humans and reaches reported blood levels of up to 140 µg/ml, much higher than the fungicidal concentrations, which are in the low microgram per milliliter range (10). In addition, relevant for neurological infections, the drug has been used to treat cerebral helminthic infections, such as central nervous sytem (CNS) paragonomus; in one report, 24 patients were treated with bithionol and cures were reported for 22 when bithionol doses of 40 to 50 mg/kg of body weight/day were used (11). Some work has been performed regarding mechanisms of action related to mammalian toxicity at high concentrations. In mammalian tissues at higher doses, bithionol acts to slow rapidly growing cells, such as ovarian cells, and appears to target the NF-κB and mitogen-activated protein kinase signaling pathways (12). In addition, bithionol has been used to model allosteric binding of GTP to glutamate dehydrogenase in crystallographic studies (13). However, fungicidal mechanisms of bithionol that could inform the design of novel agents remain poorly understood.

While bithionol may not be an optimal chemical moiety for modern use because of its potentially DNA-reactive phenolic groups (14), identification of target enzymes of a potentially effective and relatively nontoxic drug may prove valuable for future drug development (7). In the present study, we utilized the method of drug affinity responsive target stability (DARTS) screening, whereby protein lysates are incubated in the presence or absence of drug, partially digested with protease, and drug-protected active site peptides are identified by differential mobility on SDS-PAGE gels followed by mass spectroscopy (15). This method was combined with a gene dosing strategy, whereby a whole-genomic overexpression library of the yeast *Saccharomyces cerevisiae* was used to identify genes whose overexpression resulted in drug resistance (gene dosing and drug affinity responsive target stability [GD-DARTS]). This method identified 75 protein targets via DARTS and 9 genes after overexpression. Combining these two modalities identified NAD-dependent enzymes whose activities were inhibited by bithionol. Molecular modeling, based on mammalian enzyme studies, was used to demonstrate binding pockets and relevant binding sites within the cryptococcal enzymes. Thus, these studies identified a new set of enzymes sharing a common inhibitor binding site and whose combined inhibition was associated with potent fungicidal activity.

## RESULTS

### Drug affinity responsive target stability.

**FIG 1:**
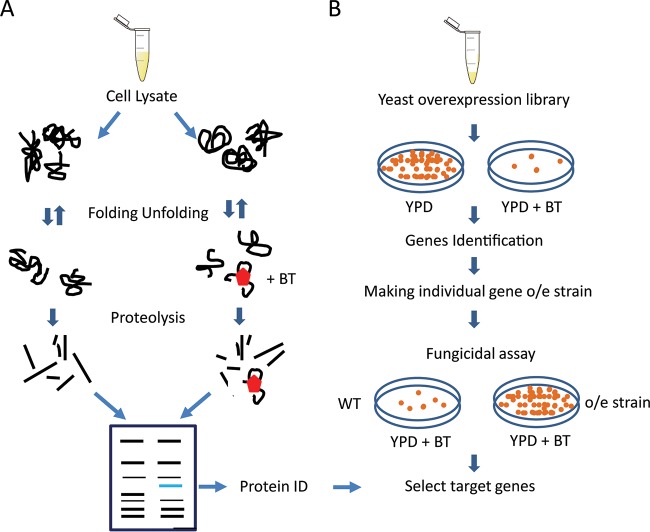
Scheme for the methodology of GD-DARTS. (A) Cell lysate is incubated in the presence (+BT) or absence of bithionol, followed by proteolysis and protein electrophoresis. Protected protein bands are excised and subjected to mass spectroscopy, as described in Materials and Methods. (B) Sensitive yeast strains are transformed with a yeast overexpression library or empty vector alone and exposed to increasing concentrations of drug, followed by recovery of resistant strains, recovery of plasmids, and sequencing of genes for identification.

**FIG 2:**
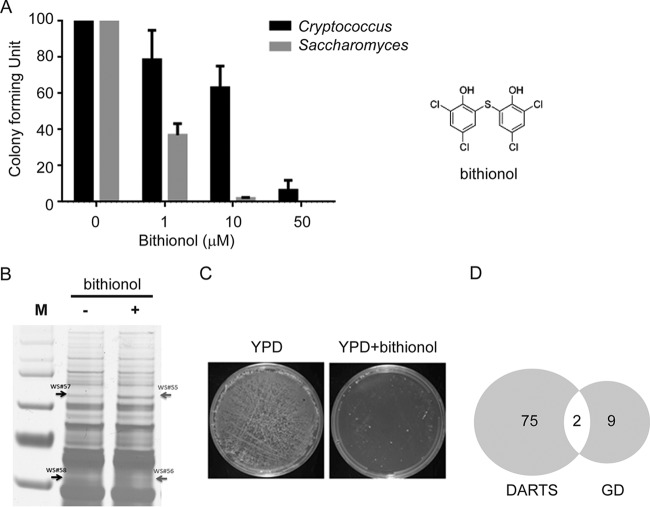
Screening results for yeast colonies resistant to bithionol. (A) Bithionol fungicidal activity against *Cryptococcus neoformans* (H99) or *Saccharomyces cerevisiae* (BY4741). Two hundred microliters of a suspension of 1,000 cells/ml in asparagine and bithionol was added to microcentrifuge tubes. Cells were then incubated at 37°C with shaking for 24 h. Samples from each tube were plated on YPD medium and incubated in a 37°C incubator for 48 h, and colonies were counted. Results are from 3 independent assays; error bars represent standard deviations. Shown on the right is the chemical structure of bithionol. (B) Target identification for potential bithionol-interacting proteins by DARTS. *S. cerevisiae* lysates were incubated with or without bithionol for 1 h. Each sample was subjected to proteolysis. The partially digested proteins were separated on a 10% bis-Tris gel and visualized with silver staining. Two specific protein bands (indicated by arrows) were further processed for mass spectrum analysis. (C) Growth on plates was compared using replica plating of a 9,000-cell mixture cultured on agar with or without 10 µM bithionol at 37°C for 2 days. (D) Venn diagram of potential target genes from the gene dosing (GD) and drug affinity responsive target stability (DARTS) screen.

The overall scheme used to identify bithionol drug targets is shown in Fig. 1. The DARTS method (Fig. 1A) utilizes fungal cell extracts incubated in the presence or absence of saturating concentrations of drug, followed by proteolysis. Drug binding peptides are essentially “protected” against proteolysis, and after gel electrophoresis they have higher motilities due to reduced rates of proteolysis compared to identical reactions in the absence of drug. The identified set of proteins derived from the retarded peptides was then compared to a gene dosing screen whereby genes were identified whose overexpression resulted in resistance to the given drug (Fig. 1B). For these studies, we utilized the genetically tractable fungus *Saccharomyces cerevisiae*. We first tested drug sensitivity against *S. cerevisiae* BY4741 and *Cryptococcus neoformans* H99, which were both sensitive to bithionol at micromolar concentrations, with increased sensitivity of the *S. cerevisiae* strain (Fig. 2A). In addition, we tested 10 cryptococcal strains which demonstrated low-micromolar fungicidal inhibition, even for strains demonstrating high-level resistance to other antifungals, such as fluconazole (Table 1). To identify potential bithionol-interacting proteins, we utilized DARTS against *S. cerevisiae* BY4741 as well as *C. neoformans* H99 protein extracts (Fig. 2B). Seventy-five candidate proteins that potentially interacted with bithionol were identified from the two yeast species (see Tables S2 and S3 in the supplemental material). Interestingly, a predominance of dehydrogenases (8), including NADH dehydrogenases, were identified.

**TABLE 1:** *In vitro* susceptibilities of 11 *Cryptococcus neoformans* strains to amphotericin B, fluconazole, and bithionol^a^

Strain	Etest IC_50_ (48 h)		Etest IC_50_ (72 h)		AC_50_ (μM) of bithionol
	Ampho B	Fluconazole	Ampho B	Fluconazole	
H99	0.19	12	0.25	24	0.7427
Bt68 alpha	0.094	3	0.25	4	0.4176
MRL862	0.25	>256	0.50	>256	1.4818
Bt27 alpha	0.125	4	0.50	6	1.8655
NIH157	0.094	48	0.25	128	1.8655
NIH38	0.094	2	0.125	2	2.0931
Bt90 alpha	0.25	6	0.50	12	1.177
NIH 7	0.25	2	0.38	1.5	2.3485
Bt12	0.125	8	0.19	8	0.5258
Bt 125 alpha	0.064	0.25	0.094	0.38	2.63
NIH 9	0.25	12	0.50	48	1.3207

a Abbreviations: Ampho B, amphotericin B; IC_50_, the concentration of an inhibitor where the response is reduced by half; AC_50_, half-maximal fungicidal activity concentration.

### Identification of candidate bithionol fungicidal target genes.

**FIG 3:**
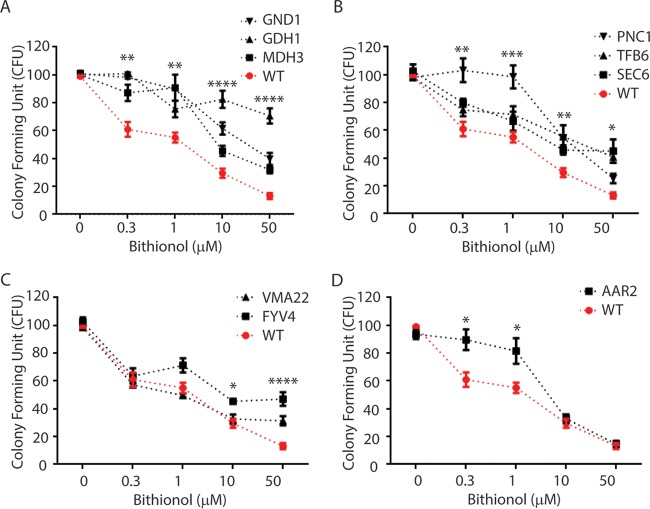
Fungicidal assay results for *S. cerevisiae* overexpression strains exposed to increasing concentrations of bithionol. The indicated overexpressed strains and the WT BY4741 strain were used, and assays to determine CFU were performed in triplicate. *, *P* < 0.05; **, *P* < 0.01; ***, *P* < 0.001; ****, *P* < 0.0001.

Because of a large number of candidate bithionol-interacting proteins identified through DARTS, a complementary gene dosing strategy was utilized to identify genes whose products conveyed resistance to bithionol. An overexpression genomic library containing 4,500 genes was transformed into the sensitive *S. cerevisiae* strain and screened in the presence of increasing amounts of bithionol. Using replica plating, we obtained bithionol-resistant *S. cerevisiae* colonies (Fig. 2C). Plasmids were recovered from resistant colonies, and genes were identified through sequencing. Because the average size of the inserts in the overexpression library was 10 kb and inserts contained multiple genes, single-gene overexpression constructs were introduced into *S. cerevisiae* to identify and confirm the gene that conveyed the resistance phenotype. Using this method, 9 genes were identified whose overexpression conferred resistance on plate assays with bithionol compared to the wild type (WT) (Fig. 2D). A summary of the corresponding functions of the protein products of the overexpressed genes are shown in Table 2. Of the 9 genes, three shared a common putative NAD-dependent dehydrogenase activity: malate dehydrogenase (*MDH3*), glutamate dehydrogenase (*GDH1*), and 6-phosphogluconate dehydrogenase (*GND1*) (Fig. 3A), and two (*GDH1* and *GND1*) were also identified in the DARTS screen, suggesting a class effect against these enzymes. In addition, yeast strains overexpressing the gene *PNC1*, *TFB6*, or *Sec6* also conferred resistance to bithionol compared to WT (Fig. 3B). Two additional genes, *VMA22* and *FYV4*, conferred increased resistance only at high concentrations of 50 µM, while they had similar sensitivities to the wild type at low concentrations of 0.3 µM (Fig. 3C). On the other hand, one gene, *AAR2*, conferred resistance only at low concentrations (0.3 and 1.0 µM), with similar sensitivities at high concentrations (10 and 50 µM) (Fig. 3D). These latter activities suggest indirect roles in bithionol resistance.

**TABLE 2:** Putative bithionol target genes of *S. cerevisiae* based on the gene dosing methodology

Gene name	Systematic name	Description
*MDH3*	YDL078c	Peroxisomal malate dehydrogenase
*GDH1*	YOR375c	NADP^+^-dependent glutamate dehydrogenase
*GND1*	YHR183w	6-Phosphogluconate dehydrogenase
*PNC1*	YGL037c	Nicotinamidase that converts nicotinamide to nicotinic acid; part of the NAD^+^ salvage pathway
*SEC6*	YIL068C	Essential 88-kDa subunit of the exocyst complex
*TFB6*	YOR352w	Subunit of TFIIH complex; facilitates dissociation of the Ssl2p helices from TFIIH
*AAR2*	YBL074C	Component of the U5 snRNP complex; required for splicing of U3 precursors
*FYV4*	YHR059w	Protein of unknown function; required for survival upon exposure to K1 killer toxin
*VMA22*	YHR060	Protein that is required for vacuolar H^+^-ATPase (V-ATPase) function

### Validation of NAD-dependent dehydrogenases as targets of bithionol.

**FIG 4:**
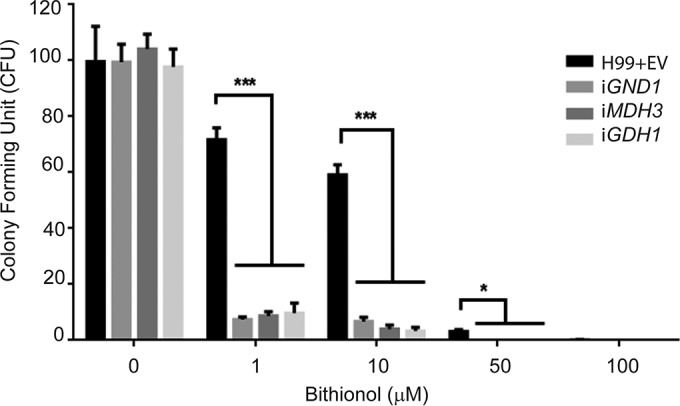
Fungicidal assay results for *C. neoformans* MDH3, *GDH1*, and *GND1* RNAi strains exposed to increasing concentrations of bithionol. Two hundred microliters of a suspension of 1,000 cells/ml of each indicated strain in medium containing asparagine and the indicated concentration of bithionol was added to a microcentrifuge tube. Cells were then incubated at 37°C with shaking for 24 h. Samples from each tube were plated on YPD medium and incubated in a 30°C incubator for 48 h, and colonies were counted. Assays were performed in triplicate. *, *P* < 0.05; ***, *P* < 0.001.

The three gene products sharing a common putative NAD-dependent dehydrogenase activity, Mdh3, Gdh1, and Gnd1, from the gene dosing assay were selected for further evaluation. All these genes are expressed under no-glucose conditions (see Fig. S1 in the supplemental material). RNA interference (RNAi) strains of *MDH3*, *GDH1*, and *GND1* were constructed to examine the effect on drug resistance to gene dosing reduction and to confirm their role in *C. neoformans*. As shown in Fig. 4, all knockdown strains exhibited increased sensitivity against bithionol compared to WT strains containing empty vector alone.

**FIG 5:**
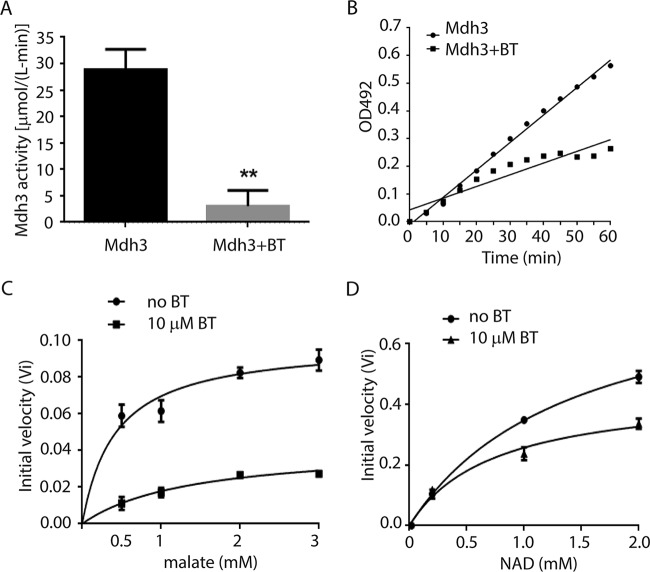
Bithionol inhibits recombinant cryptococcal Mdh3. (A and B) Assays were performed by adding 50 ng protein to 50 µl of assay solution with or without 10 µM bithionol, and mixtures were incubated at 37°C for 30 min (A), or assays were conducted at 5-min intervals at 37°C (B). Activity was measured via spectrophotometer at 492 nm using a malate dehydrogenase activity kit according to the manufacturer’s directions. (C and D) The steady-state velocities at varied glutamate and NAD concentrations in 0.1 M sodium phosphate buffer (pH 7.5) with or without 10 µM bithionol. Activity was determined based on NAD turnover at 340 nm. Assays were performed in triplicate. Error bars show standard deviations.

To further confirm NAD-dependent dehydrogenases as a direct target of bithionol, we selected the representative enzyme malate dehydrogenase (Mdh3) and expressed and purified recombinant Mdh3 in *S. cerevisiae* as a fusion protein with the affinity tag maltose binding protein (16). Recombinant cryptococcal proteins were not active when expressed in *Escherichia coli* (data not shown). Recombinant enzyme expressed in *S. cerevisiae* or cryptococcal extract was active both by a commercial assay (Fig. 5A; see also Fig. S2 in the supplemental material) and using constituent substrates (Fig. 5B), and the cryptococcal enzyme was found to require NAD for reduction of malate. In addition, bithionol inhibited Mdh3 activity in a Michaelis-Menten-type manner, displaying noncompetitive inhibition of both malate and NAD with *K_i_* inhibitory constants of 3.0 µM for malate and 18.45 µM for NAD (Fig. 5C and D). These findings suggest that bithionol acts as an allosteric inhibitor of both malate and NAD for Mdh3. Inhibition of multiple dehydrogenase targets likely contributes to the potent fungicidal activity of bithionol.

### Bithionol binds to a conserved fungal NAD-dependent dehydrogenase binding pocket.

**FIG 6:**
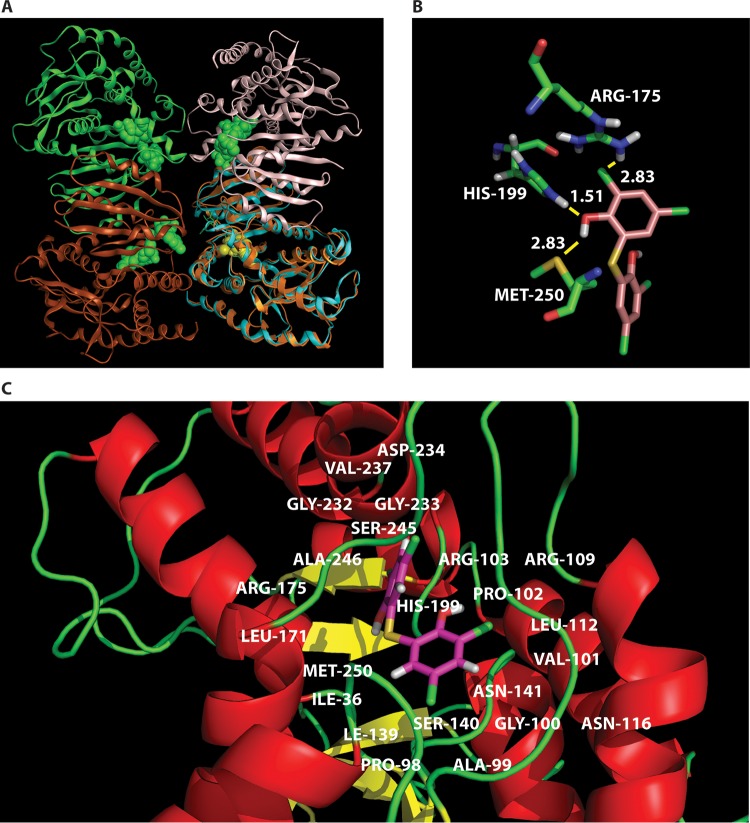
The binding environment of bithionol within the Mdh3 core region. (A and B) Ribbon (A) and stick (B) figures of the MDH-bithionol complex, with some of the contact residues highlighted. In panel B, the predicted binding energies (in kilocalories per mole) at the bithionol binding sites (yellow) are shown. (C) A closer view of the Mdh3 binding pocket structure. Bithionol is docked to the modeled *C. neoformans* Mdh3 protein. The bithionol binding sites (yellow) and NAD and malate (green) molecules complexed with Mdh3 are indicated. Residues within 5 Å of bithionol are labeled and defined as the binding pocket.

The finding of multiple NAD dehydrogenases as bithionol targets by GD-DARTS suggested a common fungal bithionol binding site within this class of enzymes. For each of these *Cryptococcus* target sequences, a BLAST search was run against Protein Data Bank (PDB) structural parameters, and the protein structures with the highest sequence homologies for each of the *Cryptococcus* targets were identified and used as templates for homology modeling of the bithionol binding pocket, as described in Materials and Methods. The modeled *C. neoformans* protein Mdh3, which demonstrated inhibition of the recombinant cryptococcal enzyme, is shown in Fig. 6A. A close-up image of the putative fungal binding site with bithionol (yellow) and NAD and malate (green) is shown complexed with Mdh3 (Fig. 6B and C). Other dehydrogenases identified in the DARTS screen were also modeled for bithionol binding, including glutamate dehydrogenase, d-lactate dehydrogenase, dihydrolipoyl dehydrogenase, aldehyde dehydrogenase, d-arabinitol 2-dehydrogenase, and NADH dehydrogenase, and are shown in Fig. S3 in the supplemental material. The best docked configuration of bithionol within each of the binding pockets was then used as a starting point for binding energy calculations.

The calculated binding energies of bithionol are given in Table 3, with lower values denoting higher binding energies. The binding energy of bithionol to Mdh3 was predicted to be −20 kcal/mol, far larger in magnitude than the binding energy of bithionol to Gdh1 (−11 kcal/mol). These results suggest that bithionol binds to the first 5 proteins, including Mdh3 and Gdh1, but does not bind strongly to the last three. The major interactions of bithionol with the Mdh3 binding pocket are shown in Fig. 6B. Important to this binding appears to be the bridging hydrogen bonding interaction between HIS-199, bithionol hydroxyl, and MET-250. In addition, ARG-175 makes electrostatic contacts with the aromatic chlorines on bithionol. Otherwise, in Gdh1, one strong hydrogen bond exists between bithionol and LYS-138 and one C-H π bond exists between bithionol and VAL-441. Bithionol is thus predicted to be a less potent inhibitor of Gdh1 than it is of Mdh3, probably because it only has one classic hydrogen bond to Gdh1, whereas in the case of Mdh3 it has two. Taken together, these results suggest that bithionol is predicted to be a broad-spectrum NAD-dependent dehydrogenase inhibitor, with more potent inhibition of Mdh3 than of Gdh1.

**TABLE 3:** Absolute binding energies of putative bithionol target dehydrogenases^a^

Protein	Accession no.^b^	Bithionol binding energy (kcal/mol)
Lactate dehydrogenase	CNAG_02644	−32
Dihydrolipoyl dehydrogenase	CNAG_07004	−31
Malate dehydrogenase	CNAG_03225	−20
Aldehyde dehydrogenase	CNAG_02377	−19
Glutamate dehydrogenase	CNAG_01577	−9
Succinate-semialdehyde dehydrogenase	CNAG_01027	22
Arabinitol 2-dehydrogenase	CNAG_02925	29
NADH dehydrogenase	CNAG_00788	52

a The magnitude of the binding energy is related to the compound’s potency.

b Accession numbers are from the Broad Institute’s *Cryptococcus neoformans* var*. grubii* H99 database.

### Reduced levels of active bithionol in mice and humans limit the suitability of bithionol as a pharmaceutical for use against *C. neoformans*.

Because of high published serum levels of bithionol (up to 30× the level of effective fungicidal activity) and use of the drug in central nervous system infections, we investigated the suitability of the compound bithionol itself in mammalian infections. As shown in Fig. S4 in the supplemental material, treatment with a single dose of 50 mg/kg in mice resulted in peak drug levels in plasma of approximately 13.2 µg/ml, with an area under the concentration-time curve (AUC) up to the last sample point (AUC_last_) of 139 µg/ml, an AUC extrapolated to infinity (AUC_INF_) of 144 µg/ml, and an area under the drug concentration moment curve up to the last sample time point (AUMC_last_) of 861 µg/ml. In the brain, there was a peak drug concentration of 1.0 µg/ml, AUC_last_ of 139 µg/ml, AUC_INF_ of 11.3 µg/ml, and AUMC_last_ of 68.9 µg/ml. Single dosing with 10 mg/kg resulted in peak plasma drug levels of approximately 4.3 µg/ml, with an AUC_last_ of 58.3 µg/ml, AUC_INF_ of 62.6 µg/ml, and an AUMC_last_ of 395 µg/ml; in this dose group, in the brain there was a peak concentration of 0.1 µg/ml, AUC_last_ of 2.4 µg/ml, and AUMC_last_ of 20.0 µg/ml. Furthermore, in a patient with refractory *C. neoformans* meningitis after receiving treatment with amphotericin B (Ambisome), dosing with bithionol at 50 mg/kg/day for 3 days resulted in trough plasma drug levels of 36.3, 46.3, and 44.9 µg/ml on days 1, 2, and 3, respectively, and cerebrospinal fluid (CSF) drug levels on day 3 1.12 µg/ml, with a simultaneous drug level of 46.2 µg/ml in the plasma, suggesting comparative levels in humans and in mice. No side effects were identified in the patient after 3 days of therapy. However, it is important to note that these findings showed that bithionol reaches significantly lower concentrations in humans than those reported in the literature (10). Further studies were conducted in mice in an intravenous model of *C. neoformans*, to determine clearance of the organism from the brain in order to compare the efficacy of bithionol to that of amphotericin B or fluconazole. As shown in Fig. S5 in the supplemental material, bithionol at 100 mg/kg was found not to be effective compared to the vehicle alone, with deaths occurring almost simultaneously. These data also suggest that the low levels achievable in blood and CSF in both mice and humans may limit the suitability of bithionol itself as an effective anticryptococcal agent, despite its favorable fungicidal and side effect profiles.

## DISCUSSION

The present study was prompted by the identification of bithionol as a potential fungicidal agent in a large repurposing screen (17) and by multiple reports for humans suggesting high serum drug levels and low toxicity (11), as well as also bithionol’s potential utility in treating CNS infections (18). The further studies described here confirmed bithionol as a potentially useful antifungal agent against *Cryptococcus neoformans* due to its antifungal effects, suggesting its utility in identifying the antifungal target of this agent, for which few data are available regarding its activity in fungi. This was particularly important for bithionol because the presence of multiple phenolic groups in the bithionol molecule as well the poor blood and brain drug concentration levels determined using modern assay methodologies have suggested that the compound itself would require significant synthetic modifications to improve bioavailability and reduce potential DNA interactions to reduce oncogenic and teratogenic potentials.

DARTS is a recently developed method for identifying potential drug targets, and it is particularly useful for compounds that have lower affinities with binding constants in the micromolar range (15). Gene dosing strategies have also been useful for identifying potential drug targets and have typically utilized either drug-induced or systematic mutations to increase drug susceptibility (19, 20). However, overexpression (rather than gene reduction) strategies can provide a similar set of candidate drug targets, but identification of resistant colonies on plates is technically more facile than identifying hypersensitive ones. In the present study, we identified 75 candidate bithionol targets, which were reduced to 2, by using the gene dosing strategy, and we identified a third target due to its similar enzymatic process. Thus, the combination strategy of GD-DARTS proved to be an efficient method of obtaining fungicidal drug targets in the present studies. The first gene, *MDH3*, is a gene that codes for malate dehydrogenase, which is involved in the glycoxylate pathway of yeast and catalyzes the oxidation of malate to oxaloacetate (21). *GDH1* codes for glutamate dehydrogenase 1, an enzyme that catalyzes the synthesis of glutamate from ammonia and α-ketoglutarate (22). *GND1* codes for 6-phosphogluconate dehydrogenase, which catalyzes the oxidative decarboxylation of 6-phosphogluconate to ribulose 5-phosphate and CO_2_, with concomitant reduction of NAD to NADH (23). Further modeling studies identified additional structurally conserved NAD dehydrogenases that were likely bithionol targets. Lactate dehydrogenase (LDH) catalyzes the interconversion of pyruvate and lactate with concomitant interconversion of NADH and NAD^+^. Replicating cells rely on increased glycolysis, resulting in increased lactate production instead of aerobic respiration in the mitochondria, even under oxygen-sufficient conditions (24). Dihydrolipoamide dehydrogenase (DLD) is a flavoprotein enzyme that oxidizes dihydrolipoamide to lipoamide. DLD is a mitochondrial enzyme that plays a vital role in energy metabolism in eukaryotes, converting dihydrolipoic acid and NAD^+^ into lipoic acid and NADH (25). Aldehyde dehydrogenases (EC 1.2.1.3) are a group of enzymes that catalyze the oxidation (dehydrogenation) of aldehydes (26). The active site of the aldehyde dehydrogenase enzyme is largely conserved throughout the different classes of the enzyme. The active site binds to one molecule of an aldehyde and an NAD^+^ that functions as a cofactor. A cysteine and a glutamate interact with the aldehyde substrate.

Allosteric inhibition was found to be important for bithionol inhibition of fungal dehydrogenases, exemplified by inhibition of the class representative, Mdh3. Modeling studies, based on crystallographic studies of the mammalian Gdh1 (13, 27), identified a shared bithionol binding site, offering an explanation for its potent fungicidal activity. Previous studies in other pathogens, such as *Trichomonas vaginalis*, have suggested that bithionol inhibits the production of volatile thiols from l-methionine (28), which could be due to inhibition of enzymes such as glutamate dehydrogenases that have been linked to methionine metabolism (29). Bithionol has also been demonstrated to inhibit respiration of intact trophozoites of *Entamoeba histolytica* (30). Since maintenance of NAD/NADH ratios is important for efficient respiration (31), it is plausible that inhibition of multiple NAD-dependent enzymes involved in alternative substrate acquisition, such as lactate dehydrogenase, could result in pathogen respiratory perturbations.

Other potential indirect targets were identified in the gene dosing study and may also play a role in bithionol fungicidal activities, but they were not further characterized in recombinant enzyme studies as they were not suggested to be targets based on the modeling studies. For example, *PNC1* is a gene that codes for nicotinamidase, a protein that converts nicotinamide into nicotinic acid. Because nicotinamidase is the first enzyme involved in the NAD^+^ salvage pathway, which functions to produce NAD^+^ (32). These results suggest that bithionol could hinder many interacting pathways involved with dehydrogenases by also inhibiting the synthesis of NAD^+^. In addition, inhibition of potential virulence factors may potentiate bithionol’s effects, such as *FYV4*, which codes for a mitochondrial protein implicated in resistance to K1 killer toxin, which binds to the cell wall and allows it to form pores (33). Genes conveying resistance to bithionol include *TFB6*, which codes for a subunit involved in the formation of the TFIIH complex and is needed to form the essential RNA polymerase II preinitiation complex (34). More specifically, Tfb6 phosphorylates Ssl2, which is another TFIIH subunit with helicase activity, and allows the two subunits to form a heterodimer that dissociates from the TFIIH (34). As an indirect mechanism, overexpression of the *TFB6* gene could allow increased transcription of multiple genes necessary to counteract bithionol’s activity. Such an indirect mechanism would explain their absence in the DART screen. In the same way, the *AAR2* gene codes for a protein that is a component of the U5 of the snRNP complex and is integral to the splicing of U3 precursors (35). The increased resistance of the *AAR2* mutant and TFIIH overexpression strains could prove protective against fungicidal activities of bithionol. Furthermore, *Sec6* was found in the gene dosing study; this gene codes for an 88-kDa subunit of the exocyst complex which is essential for the function of the complex. The Sec6 protein is important in directing post-Golgi complex secretory vesicles to appropriate locations on the cell membrane for exocytosis (36) and is important in cryptococcal pathogenesis (37). The overexpression of this gene could aid in resistance by potentially increasing the ability of cells to remove bithionol through exocytosis. On the other hand, *VMA22* is a gene that codes for an endoplasmic reticulum protein required for synthesis of vacuolar proton-transporting ATPase, which is required for creating the acidic environment of lysosomes and other organelles (38, 39). The overexpression of the *VMA22* gene could aid in providing an environment for lysosomes, endosomes, and other organelles to maintain an acidic internal environment when exposed to bithionol.

It was disappointing that bithionol itself was not a more effective anticryptococcal agent. We felt that it was important to include negative data concerning this drug for CNS cryptococcosis, both to prevent inadvertent clinical use and to address recent concerns that negative data are sometimes omitted despite their potentially sizeable scientific impact in generating new hypotheses (40). Serum and brain drug levels of approximately 40 and 1 µg/ml, respectively, corresponded to levels that produced only a 50% fungicidal activity in our assay, suggesting that ineffective absorption and transport through the blood-brain barrier was a strong contributor to this ineffectiveness in the mouse model. Previous assays estimated serum drug levels of 140 µg/ml in humans, but the methods for these assays were not described (10); however, these assays, conducted in the early 1960s, likely used an absorptive method alone after chromatography, rather than high-resolution chromatography and mass spectroscopy, which were used in the present studies. Further reductions through protein binding or problems in cellular diffusion could have further reduced effective concentrations. Removing potentially chromatin-damaging phenolic groups (41) and increasing solubility may improve the effectiveness of the agent. It is encouraging that a compound with only a single phenolic group, GW5074, was also found to bind to a similar site of mammalian enzymes (13). We did not test a pulmonary model of cryptococcosis, because there is less need for an anticryptococcal drug for pulmonary cryptococcosis, for which azole therapy is preferred (42).

In summary, the combination GD-DARTS screen yielded a powerful method of identifying potential drug targets. This method, combined with modeling studies, identified multiple NAD-dependent dehydrogenases as potential fungicidal drug targets of the model antiparasitic agent, bithionol. Further studies identifying similar NAD-dependent dehydrogenase binding compounds are likely to identify useful fungicidal candidates.

## MATERIALS AND METHODS

### Strains and growth media.

*Saccharomyces cerevisiae* strain BY4741 and *Cryptococcus neoformans* strain H99 (ATCC 208821; a kind gift of J. Perfect) were used in this study. H99-FOA was the recipient strain for the expression of RNAi constructs. Cryptococcal strains were grown in YPD (2% glucose, 1% yeast extract, 2% Bacto peptone) or asparagine salts with or without 2% glucose (as indicated) and 1 g/liter asparagine, 10 mM sodium phosphate (pH 6.5), and 0.25 g/liter MgSO_4_. *S. cerevisiae* strains were grown in SD-leu medium (drop-out mix synthetic minus leucine; catalog c13012203; U.S. Biological) with or without bithionol.

### Patient participation and study drug.

Bithionol was a generous gift of the Centers for Disease Control and Prevention under single-patient FDA investigational new drug number 118,453. 

The National Institute of Allergy and Infectious Diseases (NIAID) Institutional Review Board (IRB) approved the study under NIAID protocol 93I-0106. The subject provided written informed consent after obtaining National Institutes of Health (NIH) bioethics consultation.

### Bithionol fungicidal assays.

*S. cerevisiae* and *C. neoformans* cells were grown on YPD agar at 30°C for 48 h. Cells were counted and diluted with phosphate-buffered saline (PBS) to a final concentration of 1,000 cells/ml. Aliquots of 400 μl of each cell solution were placed into microcentrifuge tubes, and concentrations of 0, 0.3, 1, 10, and 50 µM bithionol were added. The bithionol was diluted with dimethyl sulfoxide (DMSO) to achieve its corresponding concentrations, and DMSO to 1% was additionally added to the samples without bithionol as a control. The samples were grown overnight at 30°C with shaking. After incubation, 200-µl aliquots of the suspensions were plated on YPD and incubated for 48 h at 30°C. Colonies were counted on each plate, and CFU were determined. Independent experiments were performed in triplicate.

### Determination of bithionol concentrations in female Webster mouse plasma and brain samples.

Plasma samples were placed in a 96-well plate, and preweighed frozen brain samples were placed in a 48-well plate. Samples were stored at −80°C until analysis. An ultrahigh-performance liquid chromatography–tandem mass spectrometry (UPLC-MS/MS) method was developed to determine bithionol concentrations in plasma and brain samples. Mass spectrometric analysis was performed on a Waters Xevo TQ-S triple-quadrupole instrument using electrospray ionization in negative mode with selected reaction monitoring (SRM). The SRM for bithionol was 352.9/160.9 and 352.9/194.9 at a collision energy of 24 V. The separation was performed on an Acquity BEH C_18_ column (50 by 2.1 mm, 1.7 μm) using a Waters Acquity UPLC system with a 0.6-ml/min flow rate. The column temperature was maintained at 60°C. Mobile phase A was 0.1% formic acid in water, and mobile phase B was 0.1% formic acid in acetonitrile. The UPLC gradient method was 5% B (0 to 0.1 min), 5% B to 60% B (0.1 to 0.5 min), 60% B to 95% B (0.5 to 1.5 min), 95% B (1.5 to 2.2 min), and 5% B (2.2 to 2.3 min). The total run time was 2.5 min. The brain sample was homogenized with 3 volumes of water. The calibration standards (10.0 to 20,000 ng/ml) were prepared in the control blank mouse plasma and brain homogenates. Ten microliters of plasma or 40 µl of brain homogenate was mixed with 200 µl of internal standard in acetonitrile to precipitate proteins in a 96-well plate. A 1.0-µl volume of the supernatant was injected for the UPLC-MS/MS analysis.

### Animal efficacy studies.

Virulence studies were conducted using an adaptation of a previously described intravenous mouse meningoencephalitis model (43) with 10 ND4 mice for each group and using cryptococcal strain ATCC 208821. Briefly, mice were challenged with 10^4^ CFU of *C. neoformans* (strain H99), and 3 days later therapy was started with the indicated therapy, a daily intraperitoneal injection of amphotericin B or vehicle alone in an equivalent volume; daily bithionol by oral gavage, or fluconazole by oral gavage (100 µl) twice daily. All experimental procedures involving animals were conducted under guidelines of the National Institutes of Health and protocols approved by the Institutional Animal Care Committees (IACUC) of the Intramural NIH/NIAID. Statistical comparison of survival times was made by using a log-rank comparison within GraphPad Prism 5.

### Drug affinity responsive target stability.

DARTS was performed by following a previously described method (15). Briefly, *S. cerevisiae* strain BY4741 or *Cryptococcus neoformans* (strain H99) cells were lysed with M-PER (Peirce Chemical) supplemented with protease and phosphatase inhibitors. After centrifugation at 16,000 × *g* for 20 min, the protein concentration in the supernatant was quantified with a bicinchoninic acid assay kit (Peirce Protein Biology). Proteins at 5 mg/ml were treated with 110 µM bithionol or with DMSO at 4°C overnight. The samples were treated with 0.04 mg/ml pronase (catalog number P6911; Sigma-Aldrich) for 30 min at room temperature. The digestion was stopped by adding SDS-PAGE sample loading buffer and boiling the sample at 70°C for 10 min. The samples were separated on a 10% bis-Tris gel and visualized by silver staining.

The excised gel bands were destained in a 1:1 mixture of K_3_Fe(CN)_6_ (30 mM) and Na_2_S_2_O_3_ (100 mM), dehydrated in acetonitrile, reduced with dithiothreitol (50 mM), alkylated by iodoacetamide (120 mM), and digested with sequencing-grade trypsin overnight at 37°C. The digested peptides were extracted with 1% formic acid and subjected to liquid chromatography-tandem mass spectrometry (LC-MS/MS) analysis using an Agilent 1100 nanoflow LC system coupled online with a hybrid linear ion trap–Fourier transform ion cyclotron resonance (FT-ICR) instrument (LTQ-FT; Thermo Electron, San Jose, CA). Tandem MS data were used to search the *Cryptococcus neoformans* genomic database (http://www.broadinstitute.org/annotation/genome/cryptococcus_neoformans/MultiHome.html) or the *Saccharomyces* Genome Database (by using SEQUEST). Positive hits were reevaluated by using the Scaffold program (Proteome Softwares, Inc., Portland, OR), and hits that showed ≥95% probability in Scaffold were considered significant hits.

### Overexpression library and screening of yeast colonies resistant to bithionol.

An *S. cerevisiae* overexpression library was constructed using the Yeast Genomic Tiling Collection assay’s Ready Pooled DNA (YSC5103; Thermo Scientific), which contains 4,500 transformed *S. cerevisiae* colonies. Resistant yeast colonies were identified based on persistent growth on plates containing increasing amounts of bithionol in comparison to that on plates without drug by using the replica plating method. A total of 9,000 transformants were cultured on SD-Leu medium with or without 10 µM bithionol at 30°C for 2 days. Nine drug-resistant *S. cerevisiae* colonies were recovered from plates containing 10 µM bithionol. After plasmid recovery from resistant transformants, plasmid sequencing with reference to the coding sequences and coding identifications for the *S. cerevisiae* genome (http://www.yeastgenome.org) were used to identify candidate genes whose gene products conveyed resistance to bithionol. Since genomic plasmids often contain multiple genes, candidate individual bithionol resistance genes were amplified by PCR using primer sets (see Table S1 in the supplemental material), digested with XhoI restriction enzyme, inserted into the yeast expression vector pH125 (44), and selected on SD-Leu medium.

### Construction of i*MDH3*, i*GDH1*, and i*GND1 C. neoformans* mutant strains.

The cryptococcal shuttle vector pORA-KUTAP, containing the *URA5* transformation marker, was used to effect RNAi suppression of *MDH3*, *GDH1*, and *GND1* following the method of Liu et al. (45), with modification by replacement with a 500-bp fragment of intron I of *LAC1* for the intervening region between the sense and antisense strands (producing the i*MDH3*, i*GDH1*, and i*GND1* RNAi mutant constructs, respectively). First, pORA-KUTAP, containing sequence of the *EF-1a* terminator, was digested with EcoRI and ligated simultaneously to a mixture of a XhoI-digested PCR-amplified *LAC1* intron fragment from H99 (using the primers listed in Table S1 in the supplemental material) and a second XhoI-EcoRI-digested PCR-amplified fragment of the *MDH3*, *GDH1*, or *GND1* open reading frame, respectively (using primers listed in Table S1) to produce pORA-iMDH3, pORA-iGDH1, and pORA-iGND1, respectively. The plasmids were recovered, sequences were verified, linearized with SceI, and transformed into *C. neoformans* H99 *MAT*α *ura5* (H99-FOA) cells by using electroporation by standard methods (46). An H99 *MAT*α *ura5* strain transformed with a pORA-KUTAP plasmid without the RNAi construct served as a control for *URA5* expression for *in vivo* studies.

### Expression of MDH3-MBP and GDH1-MBP recombinant proteins.

For Mdh3-MBP and Gdh1-MBP fusion protein constructs, cryptococcal genes *MDH3*, *GDH1*, and *MBP* were amplified by PCR (using primer sets listed in Table S1 in the supplemental material) and cut with BamHI and HindIII for *MDH3* and *GDH1* or with HindIII and PstI for *MBP* restriction digestion. The fragments were inserted into the pH125 vector and transformed into bacterial cells by electroporation. Indicated plasmids from *Escherichia coli* were transformed into *S. cerevisiae* cells as described above. Recombinant enzyme isolation was performed as previously described with a slight modification (16). Briefly, after seeding a 50-ml culture of *S. cerevisiae* in SD-Leu medium, cells were inoculated into 1 liter of YPD broth with shaking at 30°C until mid-log phase (optical density at 600 nm of 0.4 to 0.6). Cells were centrifuged and washed twice in column buffer (20 mM Tris-Cl [pH 7.4], 0.2 M NaCl, 1 mM EDTA, 10 mM 2-mercaptoethanol, and 1 mM NaN_3_), disrupted with glass beads by vortexing for 2 min, and centrifuged. The supernatant was applied to an amylose-agarose column (Poly-Prep chromatography columns; catalog number 731-1550; Bio-Rad). After washing with column buffer, elusion buffer containing 10 mM maltose was used to elute the protein. After dialysis with PBS, the recombinant proteins were analyzed by gel electrophoresis and stained with Coomassie blue dye.

### Enzyme activity assays and RT-PCR assays.

To measure enzyme activity, enzyme assays were performed according to the manufacturer’s protocol (catalog number E-124 for malate dehydrogenase and catalog number E-123 for glutamate dehydrogenase; Biomedical Research Service and Clinical Application, University at Buffalo). Briefly, assays were performed by addition of 50 ng protein to 50 µl of assay solution with or without 10 µM bithionol, and mixtures were incubated at 37°C for 30 min. The assay was stopped by addition of 60 µl of 3% acetic acid. Enzyme activity was determined by measuring the reduction of 2-(*p*-iodophenyl)-3(*p*-nitrophenyl)-5-phenyl tetrazolium chloride (INT) to formazan, which exhibits an absorption maximum at 492 nm, with control mixtures containing reagents alone. Reverse transcription (RT) was performed on DNase-treated RNA using the iScript kit (Bio-Rad Laboratories), according to the manufacturer’s protocol. PCRs were set up using iQ SyberGreen SuperMix (Bio-Rad Laboratories), according to the manufacturer’s protocol. Quantitative RT-PCR was performed using a Bio-Rad iCycler (MyiQ2).

### Enzyme kinetics.

The malate dehydrogenase enzyme concentration was adjusted to 100 µg/ml, and the amount of enzyme in each reaction mixture was adjusted to yield optimal steady-state velocities. All solutions were made immediately prior to use, and materials were obtained from Sigma-Aldrich. Enzyme assays were performed by monitoring reduced coenzyme production at 340 nm using a Molecular Devices FlexStation3. For the kinetic assays, reaction mixtures were prepared in triplicate in 0.1 M sodium phosphate buffer (pH 7.5) with or without 10 µM bithionol. When the malate concentration was varied, the concentration of NAD was 0.2 mM. When the NAD concentration was varied, a concentration of 2 mM malate was utilized.

### Modeling studies and predicted binding energy calculations.

For each of the *Cryptococcus* target sequences, a BLAST (47) search was run against the Protein Data Bank (PDB) (48), and protein structures with the highest sequence homologies to each of the *Cryptococcus* targets were identified and used as templates for homology modeling. The template structures are given in the Table S4 in the supplemental material. Structural models for each of the *Cryptococcus* target sequences were generated using the homology model application within the Molecular Operating Environment (MOE) program with default parameters. The binding pockets in the modeled structures were identified by aligning to the template structure. Locations of the template ligands relative to the modeled structures thereby identified model structure binding pockets.

Cryptococcal target-bithionol complex binding energies were minimized to a 0.1 RMSD (root mean square deviation) by using the MMFF94 force field (49) (within MOE). The binding energy of bithionol to the protein (ΔE_bind_) was calculated as follows: ΔE_bind_ = E_complex_ − E_protein_ − E_ligand_. The total energy of the minimized systems was calculated and labeled the E_complex_. Bithionol was then removed from each of the target proteins, and the target proteins were then minimized (without bithionol); these energies were calculated and labeled the E_protein_. Bithionol itself was minimized (starting with its protein-bound structure). The energy of minimized bithionol binding energy was labeled E_ligand_, as described in reference 47.

### MIC determinations.

Fluconazole MICs were determined via the Etest (AB Biodisk). Etests were carried out as described by the manufacturer and were performed on SD agar (1× YNB without amino acids, 2% glucose).

## SUPPLEMENTAL MATERIAL

Figure S1  Quantitative RT-PCR results for *MDH3*, *GDH1*, and *GND1* expression under no-glucose conditions. *C. neoformans* H99 cells were incubated in YPD liquid medium to mid-log phase and then transferred to asparagine salts without glucose and incubated for 3 h. Real-time RT-PCR was performed using the primer set iqMDH3, iqGDH1, and igGND1 (primers are listed in Table S1 of this supplemental material). Download Figure S1, TIF file, 0.1 MB

Figure S2  Bithionol inhibits cryptococcal dehydrogenase activity. Assays were performed by adding 50 ng of Mdh3 (A) or Gdh1 (B) to 50 µl of assay solution with or without 10 µM bithionol, and mixtures were incubated at 37°C for 30 min. Activity was measured with a spectrophotometer at 492 nm, using malate and glutamate dehydrogenase activity kits according to the manufacturer’s protocol. Assays were performed in triplicate. The extract consisted of 50 ng total protein (cryptococcal crude extract). Download Figure S2, TIF file, 1.1 MB

Figure S3  Predicted binding of bithionol to dehydrogenases. (A) GDH1 (CNAG_01577) showed major interactions of bithionol with the glutamate dehydrogenase binding pocket. One strong hydrogen bond exists between bithionol and LYS-138, and one C-H π bond exists between bithionol and VAL-441. (B) d-Lactate dehydrogenase (DLD) showed major interactions of bithionol with the DLD (CNAG_02664) binding pocket. There are two very strong hydrogen bonding interactions between bithionol and ASP-146 and ASP-342 on DLD. (C) Dihydrolipoyl dehydrogenase catalyzes major interactions of bithionol with the dihydrolipoyl dehydrogenase (CNAG_07004) binding pocket. ASP-346 makes a strong hydrogen bonding interaction with the upper hydroxyl group on bithionol, while ARG-318 makes a strong cation π interaction with the upper phenyl ring of bithionol. The lower phenyl ring of bithionol is sandwiched between LEU-365 and ILE-229, each of which makes a C-H π interaction with the lower phenyl ring. (D) Aldehyde dehydrogenase (CNAG_02377) catalyzes major interactions of bithionol with the aldehyde dehydrogenase (CNAG_07004) binding pocket. No standard hydrogen bonds exist between bithionol and this protein; however, there are multiple C-H π interactions. (E) d-Arabinitol 2-dehydrogenase NADH dehydrogenase. Bithionol is predicted to be inactive against d-arabinitol 2-dehydrogenase (CNAG_02925) and NADH dehydrogenase (CNAG_00788), mainly because the binding pocket in these cases is too small to accommodate bithionol without steric clashes. Download Figure S3, TIF file, 0.9 MB

Figure S4  Pharmacokinetcs of bithionol in mice. A single dose of bithionol at the indicated dosages was given by gavage, and serum drug levels at the indicated times were determined. Mice were also sacrificed at the indicated time intervals, and blood and brain tissues were assayed for bithionol via HPLC-MS as described in Materials and Methods in the main text. Download Figure S4, TIF file, 0.4 MB

Figure S5  Bithionol treatment in an intravenous model of cryptococcosis. Mice were challenged with 10^4^ CFU of *C. neoformans* (strain H99), and 3 days later treatment began with the indicated therapy: daily intraperitoneal injection of amphotericin B (Ampho-B) or vehicle alone in an equivalent volume, or daily bithionol by oral gavage or fluconazole (Fluc) by oral gavage twice daily. Download Figure S5, TIF file, 0.2 MB

Table S1  Primers used in this study. Table S1, DOCX file, 0.02 MB

Table S2  Potential bithionol-interacting proteins of *S. cerevisiae* by drug affinity responsive target stability and mass spectrometry analysis. Table S2, DOCX file, 0.02 MB

Table S3  Potential bithionol-interacting proteins of *C. neoformans*, based on drug affinity responsive target stability and mass spectrometry analysis. Table S3, DOCX file, 0.01 MB

Table S4  PDB IDs of structural templates used to model selected CNAG proteins. Table S4, DOCX file, 0.01 MB
