# Supplementary figures and images for: Identification of Multiple Cryptococcal Fungicidal Drug Targets by Combined Gene Dosing and Drug Affinity Responsive Target Stability Screening
Source: mBio. 2016 Aug 2;7(4):e01073-16. doi: 10.1128/mBio.01073-16 (PMC4981720; doi:10.1128/mBio.01073-16)

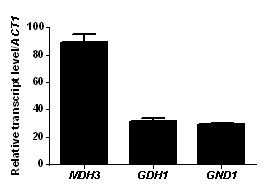

Supplement: Figure S1 — Quantitative RT-PCR results for MDH3, GDH1, and GND1 expression under no-glucose conditions. C. neoformans H99 cells were incubated in YPD liquid medium to mid-log phase and then transferred to asparagine salts without glucose and incubated for 3 h. Real-time RT-PCR was performed using the primer set iqMDH3, iqGDH1, and igGND1 (primers are listed in Table S1 of this supplemental material). Download [file mbo004162903sf1.tif]

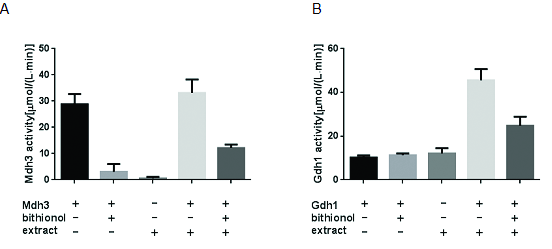

Supplement: Figure S2 — Bithionol inhibits cryptococcal dehydrogenase activity. Assays were performed by adding 50 ng of Mdh3 (A) or Gdh1 (B) to 50 µl of assay solution with or without 10 µM bithionol, and mixtures were incubated at 37°C for 30 min. Activity was measured with a spectrophotometer at 492 nm, using malate and glutamate dehydrogenase activity kits according to the manufacturer’s protocol. Assays were performed in triplicate. The extract consisted of 50 ng total protein (cryptococcal crude extract). Download [file mbo004162903sf2.tif]

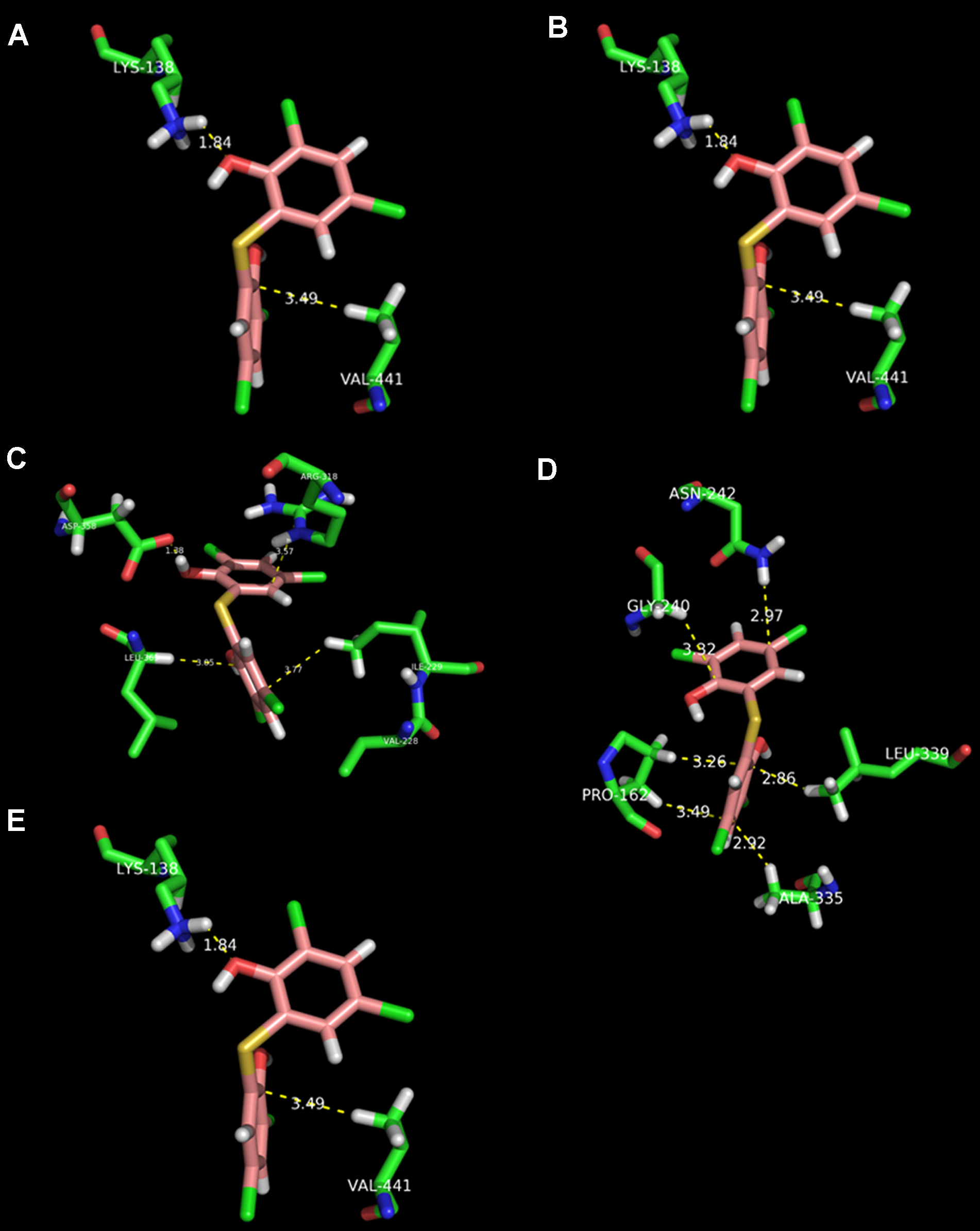

Supplement: Figure S3 — Predicted binding of bithionol to dehydrogenases. (A) GDH1 (CNAG_01577) showed major interactions of bithionol with the glutamate dehydrogenase binding pocket. One strong hydrogen bond exists between bithionol and LYS-138, and one C-H π bond exists between bithionol and VAL-441. (B) d-Lactate dehydrogenase (DLD) showed major interactions of bithionol with the DLD (CNAG_02664) binding pocket. There are two very strong hydrogen bonding interactions between bithionol and ASP-146 and ASP-342 on DLD. (C) Dihydrolipoyl dehydrogenase catalyzes major interactions of bithionol with the dihydrolipoyl dehydrogenase (CNAG_07004) binding pocket. ASP-346 makes a strong hydrogen bonding interaction with the upper hydroxyl group on bithionol, while ARG-318 makes a strong cation π interaction with the upper phenyl ring of bithionol. The lower phenyl ring of bithionol is sandwiched between LEU-365 and ILE-229, each of which makes a C-H π interaction with the lower phenyl ring. (D) Aldehyde dehydrogenase (CNAG_02377) catalyzes major interactions of bithionol with the aldehyde dehydrogenase (CNAG_07004) binding pocket. No standard hydrogen bonds exist between bithionol and this protein; however, there are multiple C-H π interactions. (E) d-Arabinitol 2-dehydrogenase NADH dehydrogenase. Bithionol is predicted to be inactive against d-arabinitol 2-dehydrogenase (CNAG_02925) and NADH dehydrogenase (CNAG_00788), mainly because the binding pocket in these cases is too small to accommodate bithionol without steric clashes. Download [file mbo004162903sf3.tif]

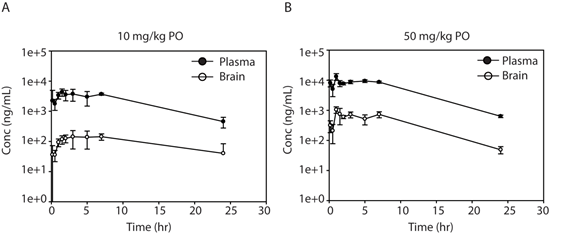

Supplement: Figure S4 — Pharmacokinetcs of bithionol in mice. A single dose of bithionol at the indicated dosages was given by gavage, and serum drug levels at the indicated times were determined. Mice were also sacrificed at the indicated time intervals, and blood and brain tissues were assayed for bithionol via HPLC-MS as described in Materials and Methods in the main text. Download [file mbo004162903sf4.tif]

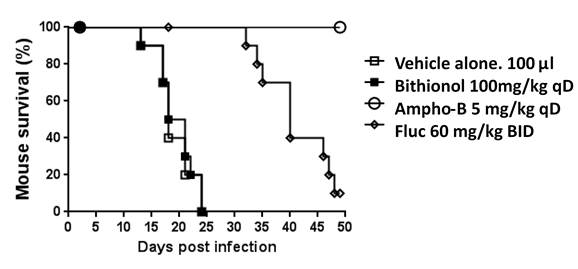

Supplement: Figure S5 — Bithionol treatment in an intravenous model of cryptococcosis. Mice were challenged with 104 CFU of C. neoformans (strain H99), and 3 days later treatment began with the indicated therapy: daily intraperitoneal injection of amphotericin B (Ampho-B) or vehicle alone in an equivalent volume, or daily bithionol by oral gavage or fluconazole (Fluc) by oral gavage twice daily. Download [file mbo004162903sf5.tif]
