# Supplementary material for: Identification of Multiple Cryptococcal Fungicidal Drug Targets by Combined Gene Dosing and Drug Affinity Responsive Target Stability Screening
Source: mBio. 2016 Aug 2;7(4):e01073-16. doi: 10.1128/mBio.01073-16 (PMC4981720; doi:10.1128/mBio.01073-16)
Supplement: Table S1 — Primers used in this study. [file mbo004162903st1.docx]

**Supplementary Table S1**. Primers used in this study.
