# Supplementary material for: Identification of Multiple Cryptococcal Fungicidal Drug Targets by Combined Gene Dosing and Drug Affinity Responsive Target Stability Screening
Source: mBio. 2016 Aug 2;7(4):e01073-16. doi: 10.1128/mBio.01073-16 (PMC4981720; doi:10.1128/mBio.01073-16)
Supplement: Table S2 — Potential bithionol-interacting proteins of S. cerevisiae by drug affinity responsive target stability and mass spectrometry analysis. [file mbo004162903st2.docx]

**Supplementary Table S2**. Potential bithionol interacting proteins of *S. cerevisiae* by drug affinity responsive target stability and mass spectrometry analysis.

| **Accession Number** | **Identified Proteins** | **Molecular Weight** |
| --- | --- | --- |
| YOR375c_BY4741 | NADP(+)-dependent glutamate dehydrogenase; synthesizes glutamate from ammonia and alpha-ketoglutarate; rate of alpha-ketoglutarate utilization differs from Gdh3p; expression regulated by nitrogen and carbon sources; GDH1 has a paralog, GDH3, that arose from the whole genome duplication | 50 kDa |
| YHR183w_BY4741 | 6-phosphogluconate dehydrogenase (decarboxylating); catalyzes an NADPH regenerating reaction in the pentose phosphate pathway; required for growth on D-glucono-delta-lactone and adaptation to oxidative stress; GND1 has a paralog, GND2, that arose from the whole genome duplication | 54 kDa |
| YFL018C_BY4741 | Dihydrolipoamide dehydrogenase, the lipoamide dehydrogenase component (E3) of the pyruvate dehydrogenase and 2-oxoglutarate dehydrogenase multi-enzyme complexes | 54 kDa |
| YBR006W_BY4741 | Succinate semialdehyde dehydrogenase involved in the utilization of gamma-aminobutyrate (GABA) as a nitrogen source; part of the 4-aminobutyrate and glutamate degradation pathways; localized to the cytoplasm | 54 kDa |
| YEL071W_BY4741 | D-lactate dehydrogenase, part of the retrograde regulon which consists of genes whose expression is stimulated by damage to mitochondria and reduced in cells grown with glutamate as the sole nitrogen source, located in the cytoplasm | 55 kDa |
| YDL171C_BY4741 | NAD(+)-dependent glutamate synthase (GOGAT), synthesizes glutamate from glutamine and alpha-ketoglutarate; with Gln1p, forms the secondary pathway for glutamate biosynthesis from ammonia; expression regulated by nitrogen source | 238 kDa |
| YHR047C_BY4741 | Arginine/alanine aminopeptidase, overproduction stimulates glycogen accumulation | 98 kDa |
| YMR125W_BY4741 | Large subunit of the nuclear mRNA cap-binding protein complex, interacts with Npl3p to carry nuclear poly(A)+ mRNA to cytoplasm; also involved in nuclear mRNA degradation and telomere maintenance; orthologous to mammalian CBP80 | 100 kDa |
| YGL207W_BY4741 | Subunit of the heterodimeric FACT complex (Spt16p-Pob3p), which associates with chromatin via interaction with Nhp6Ap and Nhp6Bp, and reorganizes nucleosomes to facilitate access to DNA by RNA and DNA polymerases | 119 kDa |
| YLR222C_BY4741 | Nucleolar protein, component of the small subunit (SSU) processome containing the U3 snoRNA that is involved in processing of pre-18S rRNA | 91 kDa |
| YMR229C_BY4741 | RNA binding protein with preference for single stranded tracts of U's involved in synthesis of both 18S and 5.8S rRNAs; component of both the ribosomal small subunit (SSU) processosome and the 90S preribosome | 193 kDa |
| YGR116W_BY4741 | Nucleosome remodeling protein; functions in various aspects of transcription, chromatin maintenance, and RNA processing; required for the maintenance of chromatin structure during transcription in order to inhibit transcription from promoters within the coding region | 168 kDa |
| YBR115C_BY4741 | Alpha aminoadipate reductase, catalyzes the reduction of alpha-aminoadipate to alpha-aminoadipate 6-semialdehyde, which is the fifth step in biosynthesis of lysine; activation requires posttranslational phosphopantetheinylation by Lys5p | 155 kDa |
| YMR076C_BY4741 | Protein required for establishment and maintenance of sister chromatid condensation and cohesion, colocalizes with cohesin on chromosomes, may function as a protein-protein interaction scaffold; also required during meiosis | 147 kDa |
| YFR002W_BY4741 | Component of the nuclear pore complex, required for nuclear pore formation; forms a subcomplex with Nsp1p, Nup57p, and Nup49p | 96 kDa |
| YGL253W_BY4741 | Hexokinase isoenzyme 2 that catalyzes phosphorylation of glucose in the cytosol; predominant hexokinase during growth on glucose; functions in the nucleus to repress expression of HXK1 and GLK1 and to induce expression of its own gene | 54 kDa |
| YPL091W_BY4741 | Cytosolic and mitochondrial glutathione oxidoreductase, converts oxidized glutathione to reduced glutathione; mitochondrial but not cytosolic form has a role in resistance to hyperoxia | 53 kDa |
| YER110C_BY4741 | Karyopherin beta, mediates nuclear import of ribosomal proteins prior to assembly into ribosomes and import of histones H3 and H4; localizes to the nuclear pore, nucleus, and cytoplasm; exhibits genetic interactions with RAI1 | 123 kDa |
| YDR190C_BY4741 | ATP-dependent DNA helicase, also known as pontin; member of the AAA+ and RuvB-like protein families; similar to Rvb2p; conserved component of multiple complexes including the INO80 complex, the Swr1 complex, and the R2TP complex (Rvb1-Rvb2-Tah1-Pih1); involved in multiple processes such as chromatin remodeling, box C/D snoRNP assembly, and RNA polymerase II assembly | 50 kDa |
| YJR121W_BY4741 | Beta subunit of the F1 sector of mitochondrial F1F0 ATP synthase, which is a large, evolutionarily conserved enzyme complex required for ATP synthesis; phosphorylated | 55 kDa |
| YPL160W_BY4741 | Cytosolic leucyl tRNA synthetase, ligates leucine to the appropriate tRNA | 124 kDa |
| YLR359W_BY4741 | Adenylosuccinate lyase, catalyzes two steps in the 'de novo' purine nucleotide biosynthetic pathway; expression is repressed by adenine and activated by Bas1p and Pho2p; mutations in human ortholog ADSL cause adenylosuccinase deficiency | 55 kDa |
| YLR300W_BY4741 | Major exo-1,3-beta-glucanase of the cell wall, involved in cell wall beta-glucan assembly; exists as three differentially glycosylated isoenzymes | 51 kDa |
| YMR012W_BY4741 | eIF3 component of unknown function; deletion causes defects in mitochondrial organization but not in growth or translation initiation, can rescue cytokinesis and mitochondrial organization defects of the Dictyostelium cluA- mutant | 145 kDa |
| YDR127W_BY4741 | Pentafunctional arom protein, catalyzes steps 2 through 6 in the biosynthesis of chorismate, which is a precursor to aromatic amino acids | 175 kDa |
| YKL035W_BY4741 | UDP-glucose pyrophosphorylase (UGPase), catalyses the reversible formation of UDP-Glc from glucose 1-phosphate and UTP, involved in a wide variety of metabolic pathways, expression modulated by Pho85p through Pho4p | 56 kDa |
| YGR124W_BY4741 | Asparagine synthetase, isozyme of Asn1p; catalyzes the synthesis of L-asparagine from L-aspartate in the asparagine biosynthetic pathway | 65 kDa |
| YPL235W_BY4741 | ATP-dependent DNA helicase, also known as reptin; member of the AAA+ and RuvB protein families, similar to Rvb1p; conserved component of multiple complexes including the INO80 complex, the Swr1 complex, and the R2TP complex (Rvb1-Rvb2-Tah1-Pih1); involved in multiple processes such as chromatin remodeling, box C/D snoRNP assembly, and RNA polymerase II assembly | 52 kDa |
| YCR053W_BY4741 | Threonine synthase, conserved protein that catalyzes formation of threonine from O-phosphohomoserine; expression is regulated by the GCN4-mediated general amino acid control pathway | 57 kDa |
| YHR020W_BY4741 | Protein of unknown function that may interact with ribosomes, based on co-purification experiments; has similarity to proline-tRNA ligase; YHR020W is an essential gene | 77 kDa |
| YER165W_BY4741 | Poly(A) binding protein, part of the 3'-end RNA-processing complex, mediates interactions between the 5' cap structure and the 3' mRNA poly(A) tail, involved in control of poly(A) tail length, interacts with translation factor eIF-4G | 64 kDa |
| YDL084W_BY4741 | Component of the TREX complex required for nuclear mRNA export; member of the DEAD-box RNA helicase superfamily and is involved in early and late steps of spliceosome assembly; homolog of the human splicing factor hUAP56 | 50 kDa |
| YOL058W_BY4741 | Arginosuccinate synthetase, catalyzes the formation of L-argininosuccinate from citrulline and L-aspartate in the arginine biosynthesis pathway; potential Cdc28p substrate | 47 kDa |
| YKL103C_BY4741 | Vacuolar aminopeptidase yscI; zinc metalloproteinase that belongs to the peptidase family M18; often used as a marker protein in studies of autophagy and cytosol to vacuole targeting (CVT) pathway | 57 kDa |
| YBR126C_BY4741 | Synthase subunit of trehalose-6-phosphate synthase/phosphatase complex, which synthesizes the storage carbohydrate trehalose; also found in a monomeric form; expression is induced by the stress response and repressed by the Ras-cAMP pathway | 56 kDa |
| YJL080C_BY4741 | Essential RNA-binding G protein effector of mating response pathway, mainly associated with nuclear envelope and ER, interacts in mRNA-dependent manner with translating ribosomes via multiple KH domains, similar to vertebrate vigilins | 135 kDa |
| YDL140C_BY4741 | RNA polymerase II largest subunit B220, part of central core; phosphorylation of C-terminal heptapeptide repeat domain regulates association with transcription and splicing factors; similar to bacterial beta-prime | 192 kDa |
| YMR309C_BY4741 | eIF3c subunit of the eukaryotic translation initiation factor 3 (eIF3), involved in the assembly of preinitiation complex and start codon selection | 93 kDa |
| YLL018C_BY4741 | Aspartyl-tRNA synthetase, primarily cytoplasmic; homodimeric enzyme that catalyzes the specific aspartylation of tRNA(Asp); class II aminoacyl tRNA synthetase; binding to its own mRNA may confer autoregulation | 64 kDa |
| YDR172W_BY4741 | Translation termination factor eRF3, has a role in mRNA deadenylation and decay; altered protein conformation creates the [PSI(+)] prion that alters translational fidelity and results in a nonsense suppressor phenotype | 77 kDa |
| YMR290C_BY4741 | ATP-dependent RNA helicase; localizes to both the nuclear periphery and nucleolus; highly enriched in nuclear pore complex fractions; constituent of 66S pre-ribosomal particles | 57 kDa |
| YKL060C_BY4741 | Fructose 1,6-bisphosphate aldolase, required for glycolysis and gluconeogenesis; catalyzes conversion of fructose 1,6 bisphosphate to glyceraldehyde-3-P and dihydroxyacetone-P; locates to mitochondrial outer surface upon oxidative stress | 40 kDa |
| YKR001C_BY4741 | Dynamin-like GTPase required for vacuolar sorting; also involved in actin cytoskeleton organization, endocytosis, late Golgi-retention of some proteins, regulation of peroxisome biogenesis | 79 kDa |
| YPL032C_BY4741 | Protein of unknown function, mutant phenotype suggests a potential role in vacuolar function; green fluorescent protein (GFP)-fusion protein localizes to the cell periphery, cytoplasm, bud, and bud neck | 92 kDa |
| YKR018C_BY4741 | Putative protein of unknown function; green fluorescent protein (GFP)-fusion protein localizes to the cytoplasm and nucleus | 82 kDa |
| YDL126C_BY4741 | ATPase involved in ubiquitin-mediated protein degradation; Cdc48p-Npl4p-Ufd1p complex participates in ER-associated degradation (ERAD) while Cdc48p-Npl4p-Vms1p complex participates in mitochondria-associated degradation (MAD); along with Npl4-Ufd1 complex, important for maintaining the cell wall integrity during heat stress to allow G1 progression; controls the proteasome-mediated degradation of Sec23p; mediates UV-induced degradation of ubiquitinated chromatin-bound Rpb1p | 92 kDa |
| YGR204W_BY4741 | Cytoplasmic trifunctional enzyme C1-tetrahydrofolate synthase, involved in single carbon metabolism and required for biosynthesis of purines, thymidylate, methionine, and histidine; null mutation causes auxotrophy for adenine and histidine | 102 kDa |
| YGR061C_BY4741 | Formylglycinamidine-ribonucleotide (FGAM)-synthetase, catalyzes a step in the 'de novo' purine nucleotide biosynthetic pathway | 149 kDa |
| YNL287W_BY4741 | Gamma subunit of coatomer, a heptameric protein complex that together with Arf1p forms the COPI coat; involved in ER to Golgi transport of selective cargo | 105 kDa |
| YJL034W_BY4741 | ATPase involved in protein import into the ER, also acts as a chaperone to mediate protein folding in the ER and may play a role in ER export of soluble proteins; regulates the unfolded protein response via interaction with Ire1p | 74 kDa |
| YMR004W_BY4741 | Protein required for sorting proteins to the vacuole; overproduction of Mvp1p suppresses several dominant VPS1 mutations; Mvp1p and Vps1p act in concert to promote membrane traffic to the vacuole; participates in transcription initiation and/or early elongation of specific genes; interacts with "foot domain" of RNA polymerase II; deletion results in abnormal CTD-Ser5 phosphorylation of RNA polymerase II at specific promoter regions | 60 kDa |
| YLR429W_BY4741 | Coronin, cortical actin cytoskeletal component that associates with the Arp2p/Arp3p complex to regulate its activity; plays a role in regulation of actin patch assembly | 73 kDa |
| YMR220W_BY4741 | Phosphomevalonate kinase, an essential cytosolic enzyme that acts in the biosynthesis of isoprenoids and sterols, including ergosterol, from mevalonate | 50 kDa |
| YKL104C_BY4741 | Glutamine-fructose-6-phosphate amidotransferase, catalyzes the formation of glucosamine-6-P and glutamate from fructose-6-P and glutamine in the first step of chitin biosynthesis | 80 kDa |
| YOR310C_BY4741 | Protein involved in pre-rRNA processing, 18S rRNA synthesis, and snoRNA synthesis; component of the small subunit processome complex, which is required for processing of pre-18S rRNA | 57 kDa |
| YLR028C_BY4741 | Enzyme of 'de novo' purine biosynthesis containing both 5-aminoimidazole-4-carboxamide ribonucleotide transformylase and inosine monophosphate cyclohydrolase activities, isozyme of Ade17p; ade16 ade17 mutants require adenine and histidine | 65 kDa |
| YER025W_BY4741 | Gamma subunit of the translation initiation factor eIF2, involved in the identification of the start codon; binds GTP when forming the ternary complex with GTP and tRNAi-Met | 58 kDa |
| YOR317W_BY4741 | Long chain fatty acyl-CoA synthetase, activates imported fatty acids with a preference for C12:0-C16:0 chain lengths; functions in long chain fatty acid import; accounts for most acyl-CoA synthetase activity; localized to lipid particles | 78 kDa |
| YLR175W_BY4741 | Pseudouridine synthase catalytic subunit of box H/ACA small nucleolar ribonucleoprotein particles (snoRNPs), acts on both large and small rRNAs and on snRNA U2; mutations in human ortholog dyskerin cause the disorder dyskeratosis congenita | 55 kDa |
| YDR012W_BY4741 | Protein component of the large (60S) ribosomal subunit, nearly identical to Rpl4Ap and has similarity to E. coli L4 and rat L4 ribosomal proteins | 39 kDa |
| YBR272C_BY4741 | Proteasome-interacting protein involved in the assembly of the base subcomplex of the 19S proteasomal regulatory particle (RP); involved in DNA mismatch repair during slow growth; weak similarity to Msh1p; related to human 19S subunit S5b | 56 kDa |
| YDR394W_BY4741 | One of six ATPases of the 19S regulatory particle of the 26S proteasome involved in the degradation of ubiquitinated substrates; substrate of N-acetyltransferase B | 48 kDa |
| YBR121C_BY4741 | Cytoplasmic and mitochondrial glycyl-tRNA synthase that ligates glycine to the cognate anticodon bearing tRNA; transcription termination factor that may interact with the 3'-end of pre-mRNA to promote 3'-end formation | 75 kDa |
| YHR024C_BY4741 | Larger subunit of the mitochondrial processing protease (MPP), essential processing enzyme that cleaves the N-terminal targeting sequences from mitochondrially imported proteins | 53 kDa |
| YBR177C_BY4741 | Acyl-coenzymeA:ethanol O-acyltransferase that plays a minor role in medium-chain fatty acid ethyl ester biosynthesis; possesses short-chain esterase activity; localizes to lipid particles and the mitochondrial outer membrane | 51 kDa |
| YMR131C_BY4741 | Essential nuclear protein involved in early steps of ribosome biogenesis; physically interacts with the ribosomal protein Rpl3p | 57 kDa |
| YHR047C_BY4741 | Arginine/alanine aminopeptidase, overproduction stimulates glycogen accumulation | 98 kDa |
| YMR125W_BY4741 | Large subunit of the nuclear mRNA cap-binding protein complex, interacts with Npl3p to carry nuclear poly(A)+ mRNA to cytoplasm; also involved in nuclear mRNA degradation and telomere maintenance; orthologous to mammalian CBP80 | 100 kDa |
| YGL207W_BY4741 | Subunit of the heterodimeric FACT complex (Spt16p-Pob3p), which associates with chromatin via interaction with Nhp6Ap and Nhp6Bp, and reorganizes nucleosomes to facilitate access to DNA by RNA and DNA polymerases | 119 kDa |
| YLR222C_BY4741 | Nucleolar protein, component of the small subunit (SSU) processome containing the U3 snoRNA that is involved in processing of pre-18S rRNA | 91 kDa |
| YMR229C_BY4741 | RNA binding protein with preference for single stranded tracts of U's involved in synthesis of both 18S and 5.8S rRNAs; component of both the ribosomal small subunit (SSU) processosome and the 90S preribosome | 193 kDa |
| YGR116W_BY4741 | Nucleosome remodeling protein; functions in various aspects of transcription, chromatin maintenance, and RNA processing; required for the maintenance of chromatin structure during transcription in order to inhibit transcription from promoters within the coding region | 168 kDa |
| YBR115C_BY4741 | Alpha aminoadipate reductase, catalyzes the reduction of alpha-aminoadipate to alpha-aminoadipate 6-semialdehyde, which is the fifth step in biosynthesis of lysine; activation requires posttranslational phosphopantetheinylation by Lys5p | 155 kDa |
| YMR076C_BY4741 | Protein required for establishment and maintenance of sister chromatid condensation and cohesion, colocalizes with cohesin on chromosomes, may function as a protein-protein interaction scaffold; also required during meiosis | 147 kDa |
| YFR002W_BY4741 | Component of the nuclear pore complex, required for nuclear pore formation; forms a subcomplex with Nsp1p, Nup57p, and Nup49p | 96 kDa |
