# Supplementary material for: Identification of Multiple Cryptococcal Fungicidal Drug Targets by Combined Gene Dosing and Drug Affinity Responsive Target Stability Screening
Source: mBio. 2016 Aug 2;7(4):e01073-16. doi: 10.1128/mBio.01073-16 (PMC4981720; doi:10.1128/mBio.01073-16)
Supplement: Table S3 — Potential bithionol-interacting proteins of C. neoformans, based on drug affinity responsive target stability and mass spectrometry analysis. [file mbo004162903st3.docx]

**Supplementary Table S3**. Potential bithionol interacting proteins of *C. neoformans* by drug affinity responsive target stability and mass spectrometry analysis.

| Accession Number | Identified Proteins | Molecular Weight |
| --- | --- | --- |
| CNAG_00788T0 | NADH dehydrogenase | 61 kDa |
| CNAG_02925T0 | D-arabinitol 2-dehydrogenase | 39 kDa |
| CNAG_02377T0 | Aldehyde dehydrogenase | 52 kDa |
| CNAG_01912T0 | Glutamine-dependent NAD(+) synthetase synthase | 82 kDa |
| CNAG_03629T0 | NADH-ubiquinone oxidoreductase | 81 kDa |
| CNAG_03168T0 | Sulfite reductase | 113 kDa |
| CNAG_06112T0 | Carbamoyl-phosphate synthase | 127 kDa |
| CNAG_04501T0 | Anthranilate synthase component 2 | 80 kDa |
| CNAG_03742T0 | Transcription factor | 103 kDa |
| CNAG_06387T0 | Ubiquitin-protein ligase | 89 kDa |
| CNAG_01928T0 | Cytoplasmic protein | 74 kDa |
| CNAG_04441T0 | Polyadenylate-binding protein | 72 kDa |
| CNAG_05042T0 | Carnitine acetyltransferase | 84 kDa |
| CNAG_05105T0 | Eukaryotic translation initiation factor 3 subunit 6 | 71 kDa |
| CNAG_06093T0 | Sec23/Sec24 family protein | 111 kDa |
| CNAG_06754T0 | Pol II transcription elongation factor | 131 kDa |
| CNAG_03767T0 | Cohesin complex subunit psm1 | 138 kDa |
| CNAG_07373T0 | Aspartate carbamoyltransferase | 255 kDa |
| CNAG_07810T0 | Mitochondrial protein | 80 kDa |
| CNAG_00992T0 | Homocitrate synthase | 54 kDa |
| CNAG_00622T0 | Acetylornithine transaminase | 50 kDa |
| CNAG_06594T0 | Oxysterol binding protein | 45 kDa |
| CNAG_00308T0 | Gly-X carboxypeptidase | 63 kDa |
| CNAG_04364T0 | Oligosaccharyl transferase stt3 subunit | 88 kDa |
| CNAG_06432T0 | AckA | 47 kDa |
| CNAG_04485T0 | Long-chain-fatty-acid-CoA-ligase | 76 kDa |
