# Supplementary material for: Identification of Multiple Cryptococcal Fungicidal Drug Targets by Combined Gene Dosing and Drug Affinity Responsive Target Stability Screening
Source: mBio. 2016 Aug 2;7(4):e01073-16. doi: 10.1128/mBio.01073-16 (PMC4981720; doi:10.1128/mBio.01073-16)
Supplement: Table S4 — PDB IDs of structural templates used to model selected CNAG proteins. [file mbo004162903st4.docx]

**Supplementary Table S4:** PDB IDs of structural templates used to model selected CNAG proteins

| Protein | Accession No | Template structure |
| --- | --- | --- |
| lactate dehydrogenase | CNAG_02664 | 3PM9 |
| dihydrolipoyl dehydrogenase | CNAG_07004 | 1JEH |
| malate dehydrogenase | CNAG_03225 | 4WLU |
| aldehyde dehydrogenase | CNAG_02377 | 3PRL |
| glutamate dehydrogenase | CNAG_01577 | 4FCC |
| succinate-semialdehyde dehydrogenase | CNAG_01027 | 3JZ4 |
| arabinitol 2-dehydrogenase | CNAG_02925 | 1VL8 |
| NADH dehydrogenase | CNAG_00788 | 4G6G |
